# Neuroprotective Potential of Jimson Weed in Methotrexate-Induced Neurotoxicity: Insights into Anti-Oxidative, Anti-Inflammatory, and Anti-Apoptotic Mechanisms *via* Modulation of Caspase-3, Interleukin-6, and Tumor Necrosis Factor-Alpha: *In silico*

**DOI:** 10.2174/0118715303350736241220090850

**Published:** 2025-08-15

**Authors:** Esther Ugo Alum, Rajapandiyan Krishnamoorthy, Mansour K. Gatasheh, Ademola Clement Famurewa, Shanthi Subbarayan, Periyasamy Vijayalakshmi, Daniel Ejim Uti

**Affiliations:** 1 Department of Research and Publications, Kampala International University, Main Campus, P.O. Box 20000, Kampala, Uganda;; 2 Department of Food Science and Nutrition, College of Food and Agriculture Sciences, King Saud University, Riyadh, 11451, Kingdom of Saudi Arabia;; 3 Department of Biochemistry, College of Science, King Saud University, P.O.Box 2455, Riyadh, 11451, Saudi Arabia;; 4 Department of Medical Biochemistry, Faculty of Basic Medical Sciences, College of Medical Sciences, Alex Ekwueme Federal University, Ndufu-Alike, Ikwo, Ebonyi State, Nigeria;; 5 Department of Grants and Partnerships, Kampala International University, Western Campus, P.O. Box: 20000, Ishaka, Bushenyi, Uganda;; 6 PG and Research Department of Biotechnology and Bioinformatics, Holy Cross College, Trichy Tamilnadu, India;; 7 Department of Biochemistry, Faculty of Basic Medical Sciences, College of Medicine, Federal University of Health Sciences, Otukpo, Benue, State Nigeria

**Keywords:** Jimson weed, *datura*, methotrexate, neurotoxicity, neurological deficits, oxidative stress

## Abstract

**Objective:**

Methotrexate (MTX) is a drug of choice for the treatment of different types of cancers and autoimmune disorders. Despite its effectiveness, its toxicity is the major drawback of its use. It is a chemotherapy drug known to cause neurotoxicity, leading to oxidative stress, inflammation, and apoptosis in the brain. We evaluated the outcome of ethanol leaf extract of Jimson weed (ELEJW) on neurotoxicity prompted by MXT use.

**Methods:**

Forty albino rats (male) were assigned into four categories (n=10): 1=Control (5 mg/kg normal saline); 2= Extract (200 mg/kg ELEJW); 3= MXT (20 mg/kg MXT); 4= Test (200 mg/kg ELEJW + 20 mg/kg MXT). MXT was given on day 18 only (ip) while ELEJW was by gavage for 21 days. Identified compounds from the plant were docked against caspase-3, interleukin-6, and tumor necrosis factor-alpha(tnf-α), while the drug-likeness properties were investigated *in silico* using ADNETSAR and Swiss adme web servers.

**Results and Discussion:**

MXT injection caused oxidative stress, inflammation, and increased cerebral cell death in rats as evidenced by elevation in SOD, CAT, GPx, AchE, and Caspase-3 activities and also increases in MDA, NO, IL-6, and TNF- α concentrations. Intriguingly, ELEJW administration to rats reversed this trend. Jimson weed showed a significant reduction in these neurotoxic effects. Jimson weed treatment led to a decrease in oxidative stress markers, a reduction in inflammatory cytokines, and a decrease in apoptotic markers in the brain tissue of methotrexate-treated rats *via* modulation of caspase-3, interleukin-6, and tumor necrosis factor-alpha(tnf-α). The histological alterations caused by MXT were resolved as intact neuronal cells with normal glial and pyramidal cells were observed. *In silico* analysis identified two compounds 1H-Indole and 2,3-Dimethylquinolin-4(1H)-one from ELEJW with potentially strong binding affinities towards caspase-3, interleukin-6, and tumor necrosis factor-alpha(tnf-α) *via* molecular docking and expression of stable hydrogen, pi, sigma, and van der Waals’ interactions between target proteins, and screened ligands, while the drug-likeness properties of these compounds show no significant violation of Lipinski, Ghose, and Verber rules.

**Conclusion:**

Co-treatment of ELEJW with MXT overturned the oxidative stress, inflammation, and histological alterations perpetuated by MXT. Therefore, Jimson weed should be explored as a supportive therapy for the management of MXT-induced neurological derangements.

## 1 INTRODUCTION

MTX is an established antineoplastic drug. It is used individually or combined with other drugs to manage various types of cancers like acute lymphoblastic leukemia (ALL), uterine, breast, and lung cancers. MXT can also be used to manage autoimmune disorders as well as the treatment of ectopic pregnancy. It is an anti-folate whose targets are DNA synthesis enzymes, especially dihydrofolate reductase and thymidylate synthase. Therefore, MXT inhibits nucleotide synthesis in active cells which culminates in apoptotic and cell cycle arrest pathways in neoplastic and synovial fibroblast cells [1].

MXT is reported to be the drug of choice for the treatment of different types of malignancies. Despite its effectiveness, its toxicity reports account for the core downside to its use. MXT-induced neurological impairment is common, especially in infants undergoing ALL treatment as about 10-50% of the infants develop some neurological deficits [2]. It is estimated that about 50-70% of infant ALL survivors present with attention and memory deficits [3]. Common symptoms of these neurological deficits include mild to severe convulsions, stroke, encephalopathy, uncoordinated movement, meningitis, and impairment in cognitive ability [4].

The mechanisms for MXT neurotoxicity are not well-understood but are thought to be multi-factorial [5]. S-adenosyl methionine (SAM) deficiency and oxidative stress have been fingered to contribute to MTX-induced neurotoxicity [6]. Recent research has investigated the processes behind methotrexate (MTX)-induced neurotoxicity and possible protective agents. MTX induces oxidative stress, inflammation, and neuronal death *via* activating endoplasmic reticulum and mitochondrial stress [7]. It also hinders hippocampus development and memory function [8]. Numerous drugs have shown potential in alleviating MTX-induced neurotoxicity. Buspirone mitigates oxidative damage and inflammation by regulating many signaling pathways, including Nrf2/HO-1 and NF-κB [9]. Cannabidiol has anti-inflammatory and anti-apoptotic properties in the brain [7]. Dexmedetomidine promotes hippocampus neurogenesis *via* the miR-15a/ROCK-1/ERK1/2/CREB/BDNF pathway [8]. Infliximab has protective properties against methotrexate-induced nephrotoxicity by mitigating oxidative stress, inflammation, and apoptosis, while enhancing mitochondrial biogenesis [10]. These results elucidate the processes behind MTX-induced toxicity and prospective treatment strategies. SAM deficiency causes demyelination and folate level decline which leads to a decline in DNA synthesis and accumulation of homocysteine culminating in seizures and other neurologic deficits [11]. The neurotoxic and memory deficit effects of MXT are well-documented [12]. Oxidative stress arises when there is an exorbitant making of free radicals such that the endogenous antioxidants are overwhelmed. This leads to damage to lipids, proteins, nucleic acids, and other macromolecules culminating in mitochondrial dysfunction and other physiological aberrations including neurological cell damage [13]. Cerebral vulnerability to oxidative stress is accompanied by an upsurge in oxidative stress signature molecules like MDA and NO, and a decline in endogenous antioxidants like GSH [14].

Oxidative stress can also enhance the production and release of cytokines like IL-1 and TNF-alpha, which are pro-inflammatory [15]. Therefore, evaluation of oxidative stress and pro-inflammatory indicators in cerebral homogenate can give a clue on the level of neurological cell damage in MXT-intoxicated rats. Aminophylline, an adenosine antagonist, and leucovorin 24−36 hours after MTX can be used to manage MXT neurological dysfunction [16]. These drugs, however, do not completely annul MXT-induced neurotoxicity as many patients especially infants, still develop acute and sub-acute neurological deficits with long-term cognitive effects. There is, therefore, the need to seek alternative and more effective measures to combat MXT-induced neurotoxicity. Plant products used as remedies for several diseases are a global antiquated habit [17-19]. The bioactive phytocompounds inherent in plants are responsible for their reported therapeutic potentials [20-23]. Identifying such compounds in plants may be an effective strategy for decreasing the incidence and severity of cancer and other diseases and boycotting the numerous adverse effects of MXT use.

Jimson weed, botanically called *Datura stramonium* is a healing herb belonging to the Solanaceae family. Its origin stems from America. It is now present in most parts of the world [24]. It’s commonly called Jimson weed, Locoweed, Angel’s Trumpet, and Thorns apple [25]. Jimson weed has exhibited some pharmacological properties such as antioxidative, anti-inflammatory, anticancer, hypolipidemic nephroprotective, and hepatoprotective properties [26-28]. In rural areas, it treats cough, chest pain, diarrhea, infertility, asthma, epilepsy, fever, and pain. Due to its psychoactive properties, some youths add it to alcoholic beverages to boost intoxication [29]. Despite the medicinal importance, all parts of Jimson weed are said to be toxic, most especially the seed. Jimson weed has varying concentrations of phytochemicals like flavonoids, alkaloids, and terpenoids [22]. However, the alkaloids are responsible for their toxicity. Some of the alkaloids include hyoscine, atropine, scopolamine, and hyoscyamine. These compounds have strong anticholinergic properties and hence could result in severe anticholinergic toxicity and neurological disorders when Jimson weed is taken in high concentration [30]. Previous studies have reported Jimson weed's nephroprotective and hepatoprotective effects in MXT-induced renal and hepatic damages through anti-oxidative and anti-inflammatory mechanisms in rats [27, 28]. Jimson weed leaf extract exhibited anti-inflammatory potential in CCl_4_-induced liver damage by attuning oxidative stress in rats [31]. Hence, our study aims to examine the impact of Jimson weed ethanol leaf extract on MXT-induced neurotoxicity since oxidative stress and inflammation are the same key mechanisms underlying MXT-induced neurological defects. Jimson weed has been proven to ameliorate inflammation and oxidative stress in MXT-treated rats. More so, as far as we know, information on the use of Jimson weed in MTX-induced neurotoxicity is scarce. Therefore, this study explored the neuroprotective impact of the co-treatment of ethanol leaf extracts of Jimson weed with MXT using a rat model.

## 2 MATERIALS

### 2.1 Chemicals and Reagents

Distilled water, standard chemicals, and reagents were utilized in the analysis. MTX was purchased from Octovia Pharmacy, Abakaliki, Nigeria.

### 2.2 Biological Materials

Jimson weed and albino rats (Wistar, all males) were used. Matured Jimson weed leaves were harvested from a farm in Amaozara Ozizza, Ebonyi State, Nigeria. Jimson weed was authenticated accordingly (Reference No.: EBSU-H-397).

### 2.3 Management of Animals

Male albino rats (weighing 95-105 g) were procured from the University of Nigeria, Animal Science Unit. They were sheltered for seven days in the Animal House of the Biochemistry Department, at Ebonyi State University, before the experiment for acclimatization. The animals had unlimited commercial rat feed and clean tap water under (25 ± 2°C) and on a normal 12:12 hours light/dark cycle photoperiod. All protocols for the safe use of animals were adhered to stringently. The animal handling procedure adhered to the criteria described in the National Institute of Health (NIH) publication (1985) for the care and use of laboratory animals. The ethical committee of the Biochemistry Department, Ebonyi State University Abakaliki, approved this study (EBSU/BCH/ET/21/006) on 25 October 2021. The protocols subscribe to the global conventional use of animal models in research. This study was conducted and reported in accordance with Animal Research: Reporting *In Vivo* Experiments (ARRIVE) guidelines.

### 2.4 Preparation of Jimson Weed Extract

Oluduro and Aderiye [32] extraction method was employed with little modification [27]. Fresh leaves of Jimson weed were plucked, cleaned meticulously under running water, and dried under shade. Thereafter, the dried sample was pulverized using a grinder. The powdered sample obtained was preserved in a sealed dark vessel. Exactly 800 g of the powdered Jimson weed leaves were immersed in 2000 ml of 98% ethanol (48 hours; room temperature) with occasional rocking. Afterward, Muslin cloth and filter paper were used for filtration. The obtained filtrate was evaporated with a rotary evaporator. The obtained Jimson weed ethanol extract was preserved in an airtight dark vessel in a refrigerator.

## 3 EXPERIMENTAL METHOD

At the expiration of the seven days’ settling in time, the rats were arbitrarily categorized into 4 (n=10) as follows:

1: Control (5 mg/kg normal saline); 2: Extract (200 mg/kg); 3: MXT (20 mg/kg MXT); 4: Test (200 mg/kg extract + MXT).

Rats in control received 5 mg/kg of normal saline orally without MXT throughout the study time. Rats in 2 (Extract only) received the Jimson weed throughout the study time (orally). Rats in 3 were injected 20 mg/kg MXT on the 18^th^ day intra-peritoneally without extract. Lastly, those in group 4 orally had 200 mg/kg body weight of the extract plus 20 mg/kg of MXT on the 18^th^ day intra-peritoneally [27]. During this study, all rats had equal and unrestricted access to food and water.

### 3.1 Samples Collections from Rats

After completing the 21-day study, rats were fasted overnight and anesthetized with ketamine. Ketamine (2 µl/kg b.w.) was injected intra-peritoneal, and the rats soon lost consciousness, after which the rats were bled out completely *via* heart puncture, using hypodermic needles and syringes. The cranium was smoothly disengaged, and the brain was harvested. The brain was cleaned with an ice-cold saline solution, dried, and homogenized. Afterward, the earned homogenate was centrifuged at 3000 rpm for 15 mins at 4°C to get the pellets which were later discarded. The obtained homogenate was utilized to assay for all parameters examined in this experiment. The cerebral tissues were promptly fixed for histopathological studies.

### 3.2 Assaying Biochemical Parameters

#### 3.2.1 Determination of Tissue Malondialdehyde (MDA) Level

Malondialdehyde (MDA) and reduced glutathione (GSH) levels were assayed spectrophotometrically by using Ohkawa *et al.* [33] and Ellman [34] procedure, respectively. Nitric oxide (NO) was quantified using Jablonska *et al.* [35] method which is based on the Griess reaction. Estimation of superoxide dismutase (SOD), catalase, and glutathione peroxidase (GPx) were spectrophotometrically done using Alia *et al.* [36], Sinha [37], and Paglia and Valentine [38] outlined protocols. IL-6 and TNF-alpha, as well as cerebral Caspase-3 activity, were assayed in brain homogenates with ELISA kits following the instructions of the provider. Acetylcholine esterase activity was evaluated colorimetrically, as explained by Ellman *et al*. [39], while the brain tissue was properly prepared for histopathological studies.

#### 3.2.2 *In silico* Analysis of Targeted Proteins and Ligands

The virtual screening of 84 ligands obtained from literature and downloaded from the PubChem database (https://pubchem.ncbi.nlm.nih.gov/) alongside 2 standard drugs (Amifostine) and (Leucovorin (Folinic Acid))were screened against Caspase-3 PDB ID: 6X8I, IL-6/IL-6Rα (PDB ID: 5FUC), and TNF-α PDB 2AZ5 obtained from the protein data bank (https://www.rcsb.org/). The top 9 compounds were selected and redocked against Caspase-3 PDB ID: 6X8I, IL-6/IL-6Rα (PDB ID: 5FUC), and TNF-α PDB 2AZ5 *via* autodock to select the top 2 compounds with highest binding affinity towards the target proteins [40]. The nine selected compounds were chosen according to binding energy, interaction strength, and molecular docking score in a stepwise analysis. The compounds were then redocked with Autodock to verify and enhance the preliminary screening results and for the selection of the top 2 compounds which was a manageable number. The three compounds with the highest binding affinities for the target proteins were selected for comprehensive study and validation. The procedure included preliminary virtual screening, docking score evaluation, and subsequent redocking to identify the final three compounds based on binding affinity.

An open-source molecular editor, Chimera 1.14, was utilized to import structures during protein preparation. This involved the elimination of non-standard residues, energy minimization of the protein, and adding hydrogen and charges using Gastiger, rendering it suitable for the subsequent docking process. Ligands sourced from the PubChem database in Structure Data File (SDF) format were processed in PyRx 0.8 software for virtual screening. Prior to docking, they underwent minimization and conversion to AutoDock PDBQT format. In the virtual high throughput screening of 84 ligands based on binding energy scores against specific proteins (Caspase-3 PDB ID: 6X8I, IL-6/IL-6Rα PDB ID: 5FUC, and TNF-α PDB ID: 2AZ5), nine ligands Myristic Acid, 2-Palmitoylglycerol, Ergost-5-en-3-ol, Linolenic Acid, (24E)-24-N-Propylidenecholesterol, cis,cis,cis-7,10,13-Hexadecatrienal, Indole, gamma-Tocopherol, and Palmitic Acid exhibited notable docking scores against target proteins. These ligands were then selected for redocking using AutoDock MGL tool 1.5.6 based on their binding affinities. Upon redocking, two selected ligands maintained superior binding affinities. Ergost-5-en-3-ol (-6), against caspase-3, (24E)-24-N-Propylidenecholesterol (-6.9) against IL-6, and (24E)-24-N-Propylidenecholesterol (-7.1) against TNF-α. For docking with 6X8I, a grid box was established with X = 22, Y = 19, and Z = 29 values. Similarly, for IL-6 (5FUC), the grid box was configured with X = 24, Y = 20, and Z = 30, and for TNF-α (PDB 2AZ5).

#### 3.2.3 Protein-Ligands Interaction

The visualization of protein-ligand binding residues was achieved by utilizing Biovia Discovery Studio. Describing the interactions between proteins and ligands is a crucial aspect of biological research, with significant implications for health, food, and energy security.

#### 3.2.4 ADMET and/or Pharmacokinetics Predictions Analysis

The evaluation of absorption, distribution, metabolism, excretion (ADME), and pharmacokinetics predictions for the leading candidate compounds was conducted through the utilization of the ADMETSAR online web servers (http://lmmd.ecust.edu.cn/admetsar2/) and Swiss adme server (http://www.swissadme.ch/index.php). This platform employs the canonical smiles of the analyzed compounds or prospective pharmaceuticals to compute physicochemical descriptors. Additionally, it provides forecasts for ADME parameters, pharmacokinetic attributes, drug-like characteristics, and medicinal chemistry compatibility for one or more small molecules in the drug development phase.

### 3.3 Statistical Analysis

Results were represented as mean ± standard deviation of triplicate values and duly analyzed with a one-way analysis of variance plus post hoc Turkey test. *P* <0.05 was adopted as significance. SPSS and Graph Pad Prism versions 22.0 and 5.00, respectively, were used for statistical analysis.

## 4 RESULTS

### 4.1 Impacts of Jimson Weed Administration on Cerebral Oxidative Stress Indices in MTX-Mediated Neurotoxicity in Albino Rats

**Fig. (1):**
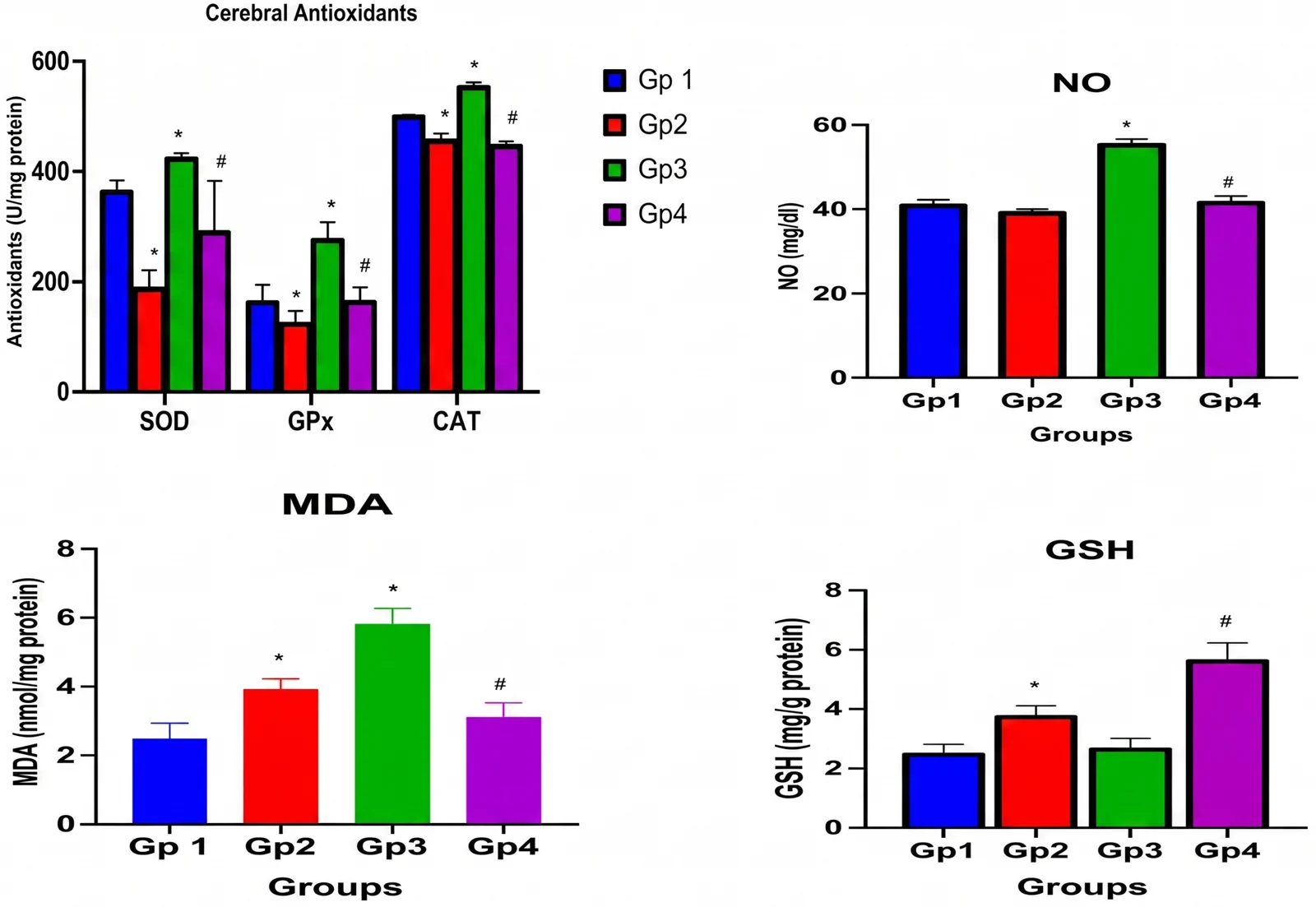
Impact of Jimson weed administration on cerebral activities of antioxidant enzymes, MDA, GSH, and NO on albino rats. Values are mean ± SD (n = 10). *P < 0.05 designates a significant difference compared to the control group. # *P* < 0.05 designates a significant difference compared to the MXT group. Gp1= Control (normal saline); Gp2= ELEJW; Gp3= MXT; Gp4= Test (ELEJW + MXT).

MXT administration created significant (*P* <0.05) elevations in cerebral SOD, CAT, and GPx activities in addition to levels of MDA and NO while level of GSH was diminished, signifying increased oxidative stress compared to normal control rats. The induction of these enzymes depicts a response to pollutant-induced oxidative stress. This was a protective mechanism. The rats’ biological system recognized the toxicant and tried to fight it by raising its arsenal of fighters (antioxidant enzymes). However, administering ethanol leaf extract of Jimson weed and MXT to rats lessened the MTX-induced cerebral dysregulations in oxidative stress indices (Fig. **1**).

### 4.2 Effect of Jimson Weed Ethanol Leaf Extract on Brain AchE and Caspase-3 Activity

**Fig. (2):**
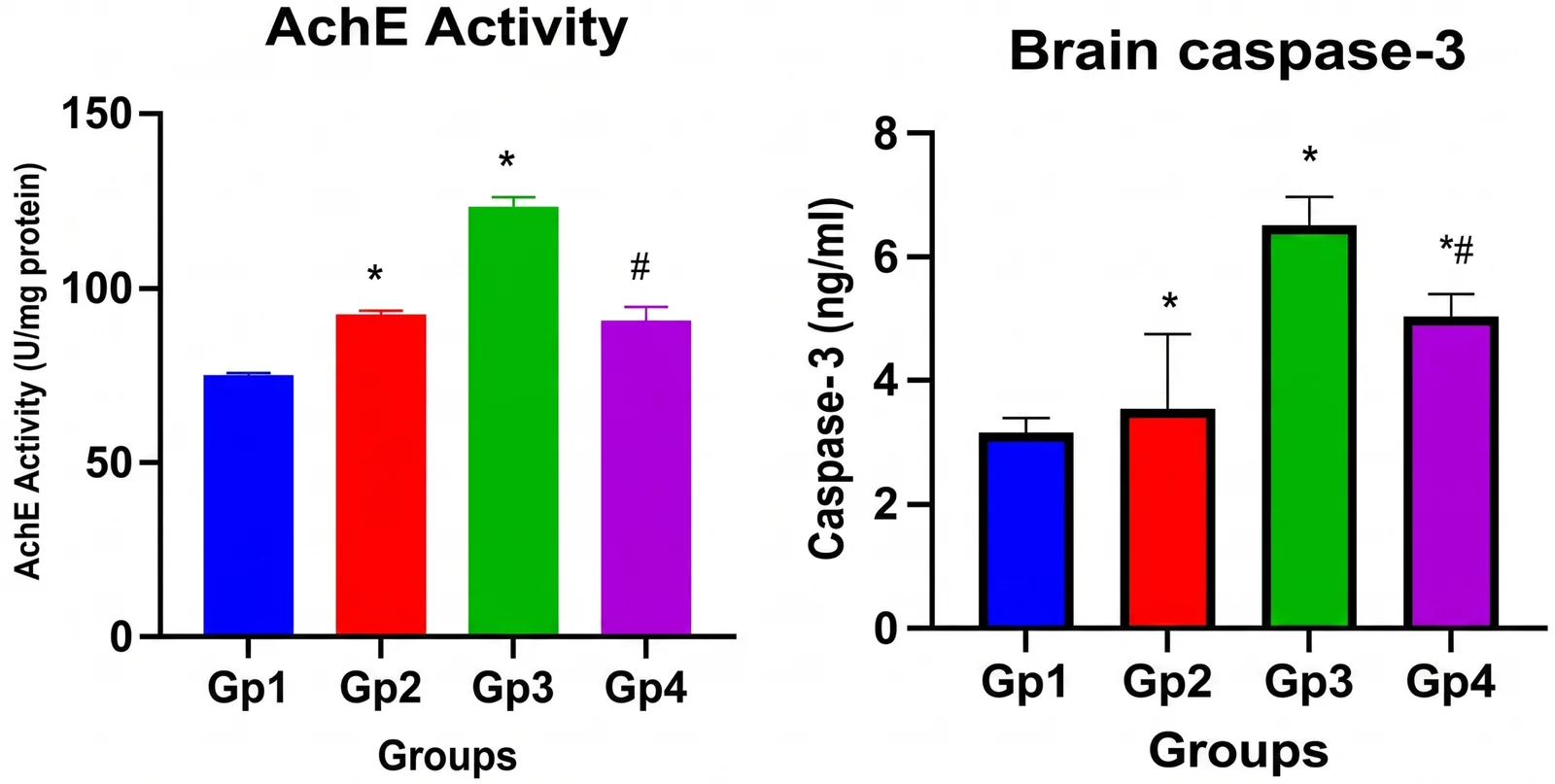
Effect of Jimson weed ethanol leaf extract and MXT on cerebral AchE and caspase-3 activities in albino rats. Values are mean ± SD (n = 10). *P < 0.05 designates a significant difference compared to the control group. # *P* < 0.05 designates a significant difference compared to the MXT group. Gp1= Control (normal saline); Gp2= ELEJW; Gp3= MXT; Gp4= Test (ELEJW + MXT).

Rats administered MXT displayed a remarkable upsurge in AchE activity compared to rats in the normal group. However, administration of ethanol leaf extract of Jimson weed and MXT to rats decreased cerebral AchE, unlike in the MXT category. Rats that received MXT exhibited elevated Caspase-3 activity, unlike the other rats. Contrarily, co-administration of ethanol leaf extract of Jimsonweed along with MXT to rats significantly decreased cerebral caspase-3 activity compared to the MXT group (Fig. **2**).

### 4.3 Effect of Jimson Weed Ethanol Leaf Extract on Some Indicators of Inflammation Markers in MTX-Triggered Neurotoxicity

**Fig. (3):**
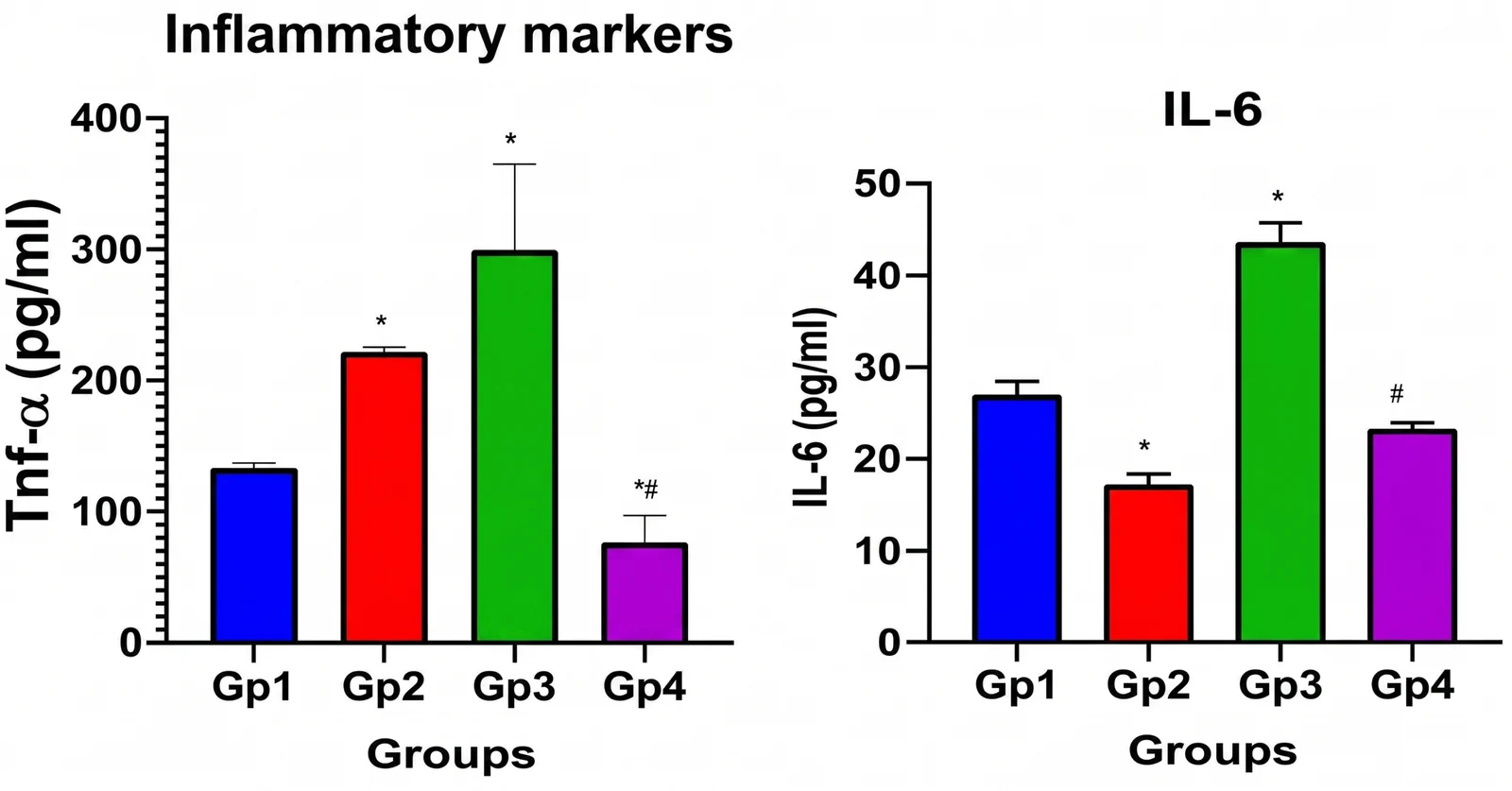
Effect of Jimson weed ethanol leaf extract on some indicators of inflammation markers in MTX-triggered neurotoxicity. Values are mean ± SD (n = 10). *P < 0.05 designates a significant difference compared to the control group. # *P* < 0.05 designates a significant difference compared to the MXT group. Gp1= Control (normal saline); Gp2= ELEJW; Gp3= MXT; Gp4= Test (ELEJW + MXT).

Injection of MTX caused significantly increased cerebral amounts of IL-6 and TNF-α in the animals. However, the ethanol leaf extract of Jimson weed attenuated this effect (Fig. **3**).

### 4.4 Effect of Jimson Weed Ethanol Leaf Extract on Histopathological Integrity of Cerebrums of MTX-induced Neurotoxicity in Rats

**Fig. (4):**
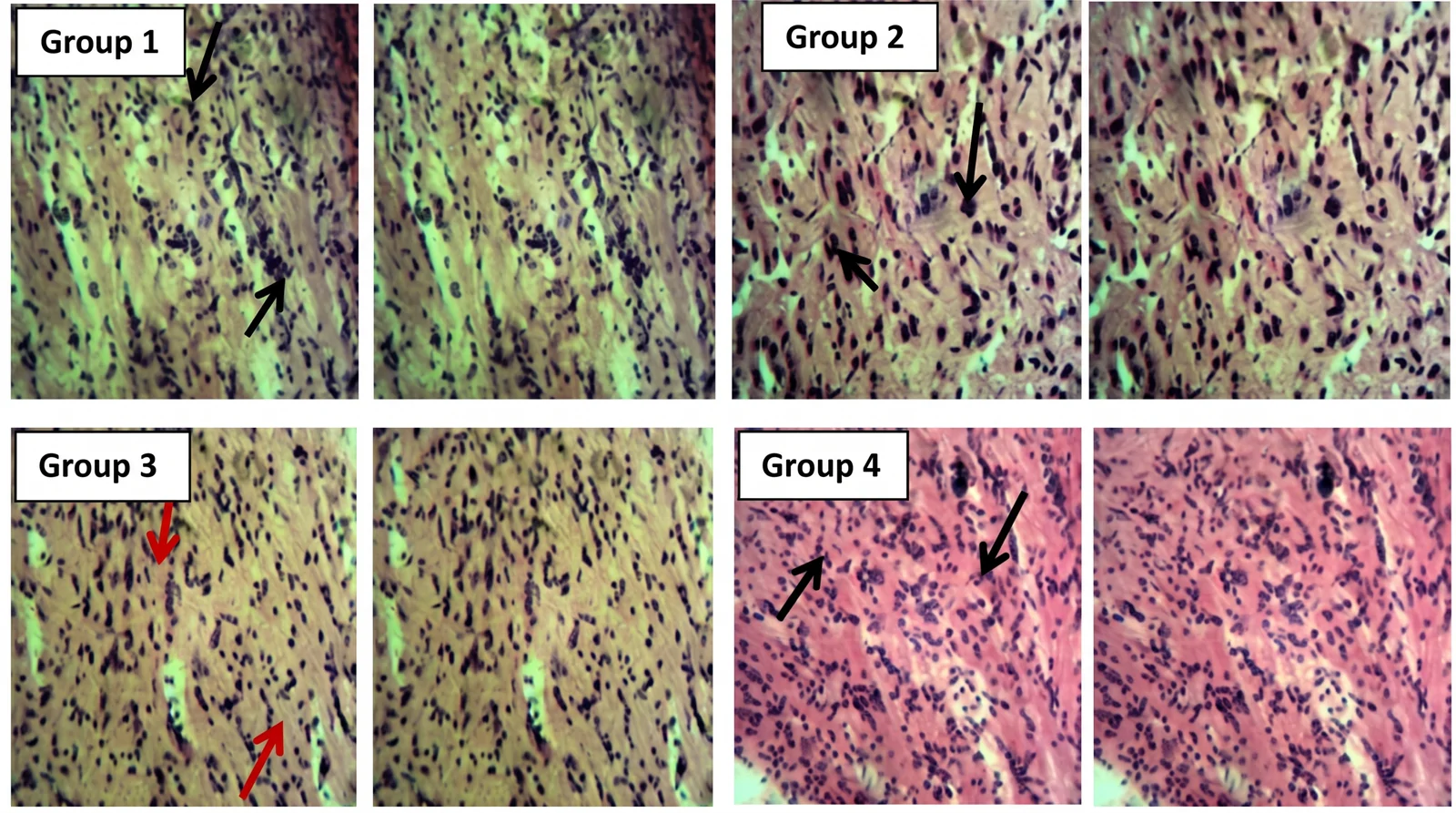
Rats in the control group displayed normal and intact neuronal cells evidenced by normal glial and pyramidal cells (black arrowheads). Rats in group 2 exhibited very mild neuronal cell changes. Rats in group 3 have neuronal cells with perceived negative histopathological alteration (red arrows). Co-administering ethanol leaf extract of Jimson weed with MXT attenuated the observed neurological damage in MXT-treated rats.

Fig. (**4**) depicts a photomicrograph representation of the brain cortex showing a high-power magnification (x400) of the prefrontal cortex showing the external granular layer (Layer 3) with their constituent granular neurons and supporting cells (black arrows). Rats in the control group displayed normal and intact neuronal cells evidenced by intact glial and pyramidal cells as depicted with black arrowheads. MXT administration caused histopathological alterations evidenced by debasement of pyramidal and glial cells, neuronal cell death, neuronophagia, and demyelination of nerve fibers, as depicted with red arrows. Interestingly, co-administration of ethanol leaf extract of Jimson weed with MXT restored the deranged neuronal cells by a marked generation of pyramidal and glial cells (Fig. **4**). Two copies of each slide have been made available; the first is edited with arrows, while the other is unedited and plain.

### 4.5 *In silico* Docking Analysis

Though all proteins had several chains, chain A of each protein was used in the docking process since the chains had identical sequences of amino acids. The results of the top nine compounds are presented in Table **1**. The following two compounds Ergost-5-en-3-ol and (24E)-24-N-Propylidenecholesterol had highest binding affinities against the three studied protein targets with results as follows; Ergost-5-en-3-ol and (-6), against caspase-3, (24E)-24-N-Propylidenecholesterol (-6.9) against IL-6, and (24E)-24-N-Propylidenecholesterol (-7.1) against TNF-α. Compared to 2 standard drugs (Amifostine) with a docked score of -6.2 against Caspase-3, and (Leucovorin (Folinic Acid)) with a docked score of -6.9, and -7.5 against IL-6 and TNF-α respectively.

The 2D and 3D diagrams of these interactions are presented in Figs. (**5A**, and **5B**), (**6A**, and **6B**), and (**7A**, and **7B**). The following binding interactions in amino acids residues were observed between Ergost-5-en-3-ol and caspase-3; LYS, GLU, ILE, PHE, THR, and LEU, while (24E)-24-N-Propylidenecholesterol (-6.9) and IL-6 had the following interactions GLU, THR, ALA, VAL, and PRO in the 2D view. The interaction of (24E)-24-N-Propylidenecholesterol (-6.9) against TNF-α showed the following amino acid residues; LYS, TYR, AL, LEU, and PHE. The 3D view indicated a strong fitting between the interacting ligand and the binding pockets of the protein.

### 4.6 Evaluation of Pharmacokinetics and Drug-Likeness Properties of Top Posed Compounds

The results in Table **2** show measured ADMET parameters for two different compounds: 1H-Indole and 2,3-Dimethylquinolin-4(1H)-one. The parameters include various molecular properties and interactions with biological targets. The parameters include molecular weight, iLogP (logarithm of the partition coefficient), hydrogen bond acceptors (HBA), hydrogen bond donors (HBD), Lipinski, Ghose, and Verber violations, as well as interactions with specific CYP enzymes and other biological factors such as Caco-2 and carcinogenicity. The results also include lead likeness violation, synthetic accessibility, TPSA (topological polar surface area), nrotb (number of rotatable bonds), and bioavailability. These parameters are important for understanding the pharmacokinetic and toxicological properties of the compounds.

## 5 DISCUSSION

Neurotoxicity is one of the drawbacks of MXT use as a remedy for cancers. Despite leucovorin use to palliate the adverse effects of MXT, about 3-15% of patients using MXT still develop neurological abnormalities like altered mental status and seizures [41]. Evidence suggests the efficacy of plant-derived natural products in managing drug-induced cerebral dysregulations [42]. Thus, medicinal plants can be explored for the management of MXT-induced neurotoxicity. As far as we know, information on the impact of Jimson weed on MTX-induced neurotoxicity is scarce. Therefore, since there is a limited report on such a study design, this study ascertained the neurologic impact of the co-treatment of ethanol leaf extracts of Jimson weed with MXT in rats.

In our research, MXT use yielded significant (*P* <0.05) elevations in cerebral SOD, CAT, and GPx activities, as well as levels of MDA and NO, while the level of GSH was depressed, signifying increased oxidative stress, unlike normal rats. However, oral administration of Jimson weed along with MXT to rats lessened the MTX-induced cerebral impairments (Fig. **1**). Increases in cerebral SOD, CAT, and GPX activities were a result of MXT intoxication. This was a protective mechanism. The rats’ biological system recognized the toxicant and tried to fight it by raising its arsenal of fighters (antioxidant enzymes). Previous studies have reported that upon exposure to pollutants, organisms usually attempt to metabolize the toxicants through their antioxidant enzymes, increasing their activities [43, 44].

Reactive oxygen species (ROS) are metabolic derivatives that aid physiological activities. However, their elevated levels signal toxicity. Their toxicity occurs when their production overwhelms the endogenous antioxidants, culminating in an oxidative stress physiological state. Escalated activities of SOD, Catalase, and GPx, plus an increase in malondialdehyde (MDA) and Nitric oxide (NO) levels, imply acute oxidative stress [14]. Substantial reports suggest oxidative stress contributes to neurological cell death (Butterfield, 2020). Therefore, the observed increase in SOD, CAT, and GPx activities, coupled with raised MDA and NO concentrations in MXT-treated animals, portend oxidative stress and consequent damage to neuronal cells. Oxidative stress can cause S-adenosylmethionine (SAM) deficiency, which has been found to contribute to MTX-induced neurotoxicity [45]. SAM deficiency causes demyelination and folate level decline, which leads to decline in DNA synthesis and accumulation of homocysteine, culminating in seizures and other neurologic deficits [12]. More so, oxidative stress causes SAM deficiency by reducing DNA methylation through DNA oxidation, increasing ten-eleven translocation (TET)- mediated hydroxymethylation, and restraining the binding of DNA methyltransferases, which produces SAM [45].

Mitochondria are the site of the electron transport chain and oxidative phosphorylation and, thus, the site of the generation of free radicals. During normal mitochondrial respiration, superoxide is formed. Superoxide dismutase (SOD) converts superoxide to hydrogen peroxide and finally into hydroxyl radicals and anions [14]. Excessive generation of superoxide such that the available SOD is not enough to convert it to water leads to a redox imbalance culminating in neurotoxicity and the yield of cytokines like IL-1 and TNF-αwhich are pro-inflammatory [16]. Thus, a boost in SOD activity can manage both oxidative stress and cytokine release. The intracellular antioxidant enzymes (SOD, CAT, and GPx) are the frontline defense during oxidative stress, while the non-enzymatic defense systems include vitamin E from diet and endogenous glutathione (GSH). These together form an array of physiological antioxidant defense systems [14]. Oxidative status plays an ample role in shaping cognitive abilities. Oxidative stress impairs cognitive behaviors and causes mood disorders [46].

Previous studies using mice have reported that cognitive impairment can be enhanced by neuronal cell death orchestrated by oxidative stress [47]. Interestingly, cognitive impairment and other related behavioral deficits can be ameliorated by antioxidant agents [48]. Therefore, the observed increase in GSH levels in Jimson weed-administered rats could probably be a result of an appreciable concentration of antioxidant compounds present in Jimson weed. Jimson weed is rich in some phytochemicals with antioxidant potential [23]. It has been proven that the intake of antioxidant-rich diets can ameliorate the severity of oxidative stress. According to Holland *et al.* [49], the intake of leafy vegetables, beans, and teas rich in flavonols reduced the severity of neuronal cell damage in Alzheimer’s disease. Therefore, antioxidant constituents present in Jimson weed could be responsible for the boost in GSH levels in the extract-treated rats.

During the inception of neurotoxicity, there is always an elevation in neuronal inducible nitric oxide synthase (iNOS), NO, and intracellular calcium ions, including a decrease in GSH concentration. In this study, we observed a similar trend in MXT-treated rats. Interestingly, co-administering ethanol leaf extract of Jimson weed reversed these orders, as seen in group 4. Reducing NO helps to prevent neuronal cell death. NO reacts with superoxide to yield peroxynitrite, which is an oxidant that propels the production of reactive radicals, which are accountable for neuronal cell damage. These reactive radicals can react with lipids, forming highly toxic MDA [42]. Therefore, arresting NO production will also go a long way in curbing the generation of MDA. Oxidative damage to lipids is called lipid peroxidation. The aftermaths of lipid peroxidation are MDA, 4-hydroxy-2-nonenal, acrolein, and isoprostanes. However, MDA is the most extensively studied because it is the most toxic to proteins and DNA and is more stable.

Systemic inflammation exacerbates neurotoxicity through microglial activation and enhanced generation and leakage of inflammatory mediators, especially cytokines. Inflammation, free radicals, and microglial activation are indispensable in the pathogenesis of many neurological complications. The Microglial is essential for central nervous system (CNS) maintenance. There is increased microglial activation during neuronal cell aging, enhanced production of ROS, and the release of prostaglandins, NO, and inflammatory cytokines [49, 50]. Inflammation and oxidative stress as a result of MXT and other chemotherapeutic agents use are widely documented in animals, including humans [28, 51]. In the present research, MXT produced a significant (*P* <0.05) increase in cytokines (TNF-*α* and IL-6) levels positing inflammation. However, co-administration of MXT with ELEJW caused a significant reduction in these cytokines. Anti-inflammatory potentials of medicinal plants are widely reported [52-54]. Therefore, the anti-inflammatory constituents inherent in Jimson weed could have exerted the observed anti-inflammatory effects noticed in the extract-treated group.

Autophagy is an intracellular cell survival apparatus in response to cellular inconvenience. It reduces intracellular ROS sources, hence cytoprotective [55]. Autophagy degrades damaged organelles and outdated proteins. Radiotherapy, chemotherapy, ROS, nutrient inadequacy, or oxygen starvation can induce autophagy. Extracellular stress signals induce apoptotic cell death by turning on the TNF-α pathway, while intracellular stress indicators, such as DNA damage, oxidative stress, or cytosolic calcium upsurge, cause apoptosis by enhancing Caspase-3 activity [56]. MTX can trigger apoptosis by enhanced generation of ROS, evidenced by increased oxidative stress. Caspase-3 activation is reported to enhance the incidence of apoptosis in neuronal cells [57]. In this study, we discovered an elevated caspase-3 activity in the MXT-treated group contrary to the control group. This corroborates previous uncoverings that investigated the neurotoxicity of MTX on hippocampal cells, cerebellum, kidney, and liver tissues [58]. Here, the administration of Jimson weed mitigated the apoptotic alterations in the cerebral cortex tissue as shown by a decline in caspase-3 activity. Recall that we observed increased cytokine levels, especially TNF-α, in MXT-treated rats. According to Zaki *et al.* [56], an upsurge in TNF-α release triggers oxidative stress and expression of caspase-3, induces apoptosis, and causes brain damage. Therefore, the TNF-α could have contributed to the over-expression of caspase-3 activity and neuronal cell death recorded in MXT-treated rats.

Jimson weed contains alkaloids such as atropine, scopolamine, and hyoscine. These alkaloids are responsible for their anticholinergic effects [22]. The alkaloids in Jimson weed act as competitive antagonists to peripheral and muscarinic acetylcholine receptors, culminating in anticholinergic syndrome [29]. This study showed that MXT remarkably elevated cerebral acetylcholine esterase (AchE) activity. This implies that the increase in cerebral activity of AchE observed in MXT-treated rats could lower the level of acetylcholine and, hence, promote neurological cell death. Our result corroborates previous studies that reported the neurodegenerative effect of neurotoxicants, evidenced by a concomitant increase in AchE activity [59]. Interestingly, co-administration of MXT with Jimson weed extract attenuated the rise in cerebral activity of AchE. According to Imran *et al.* [60], natural products can modulate AchE activity in neuro-intoxicated rats due to some bioactive agents present in natural products. Previous authors have proclaimed the anticholinergic effects of Jimson weed [61, 62]. Amazingly, an excessive decline in AchE activity is reputed to be a core toxicological apparatus of many neurotoxicants due to the conglomeration of excessive acetylcholine at synaptic cleft culminating in arousal of postsynaptic neurons and consequent neuronal death [61]. Therefore, an excessive decline in AchE activity poses neurological abnormalities. Thus, Jimson Weed's ‘two-faced’ character needs to be substantiated. Hence, maintaining equilibrium between the increase in AchE activity caused by MXT and the decrease in AchE by Jimson weed is paramount. At present, the use of therapeutic AchE inhibitors is one of the more promising strategies for the treatment of neurodegenerative disorders. AchE inhibitors enhance cholinergic neurotransmission by increasing acetylcholine levels in the brain [63]. Taken together, the combination of anti-apoptotic and AchE inhibition makes Jimson weed a promissory plant for exploration as a treatment option for MXT-induced neurological cell damage and other neurodegenerative disorders.

The present study carried out a histological examination of the cerebrum of the rats. There were well-formed, intact, consistent pyramidal neurons and minimally reactive glial cell infiltrates in the normal and Jimson weed control groups, respectively. MTX caused an acute neuronal loss in addition to obvious constricting of pyramidal neurons plus spontaneous microglial cell infiltrates. Fascinatingly, co-treatment of MXT with Jimson weed safeguarded the large pyramidal neurons, unlike rats that received MTX only, suggesting its neuroprotective potential. Our results conform with the reports of previous researchers who reported diminished neurogenesis, neuronal loss, and constricting of large pyramidal neurons in MXT-treated rats [8]. The histological findings in this study suggest that neurotoxicity emanating from MXT use perhaps caused a decline in neurogenesis. Intriguingly, co-treatment with Jimson weed palliated the altered neurological architecture caused by MXT in rats.

The results of the docking process involved three different protein targets, where the chain A of each protein was utilized due to the identical sequences of amino acids in the chains. The focus was on the top two compounds, Ergost-5-en-3-ol and (24E)-24-N-Propylidenecholesterol obtained from docking, which exhibited the highest binding affinities against the three studied protein targets: caspase-3, IL-6, and TNF-α. These findings suggest that Ergost-5-en-3-ol and (24E)-24-N-Propylidenecholesterol have promising binding affinities against caspase-3, IL-6, and TNF-α. The specific amino acid residues involved in the interactions provide insights into the potential binding sites and mechanisms of action. The strong fitting observed in the 3D view further supports the potential efficacy of these compounds as inhibitors or modulators of the studied proteins, highlighting their potential relevance in drug development or therapeutic interventions targeting these proteins.

The results in Table **2** provide a comprehensive overview of the ADMET parameters for the compounds 1H-Indole and 2,3-Dimethylquinolin-4(1H)-one. These parameters encompass a wide range of molecular properties and interactions with biological targets, shedding light on the pharmacokinetic and toxicological properties of the compounds. These parameters are crucial for assessing the compounds' drug-likeness, bioavailability, and safety profiles. By analyzing these parameters, researchers can gain insights into how the compounds may behave in biological systems, their potential interactions with specific enzymes, their permeability across biological barriers, and their likelihood of being carcinogenic. Overall, the results in Table **2** provide valuable information for researchers and drug developers to make informed decisions about these compounds' potential utility and safety in a pharmaceutical context. 1H-Indole is a naturally occurring heterocyclic compound with a bicyclic structure consisting of a benzene ring fused to a pyrrole ring [64-66]. It is a key structural element in many biologically important molecules. Some of its biological properties and roles include being a component of the amino acid tryptophan, a precursor for the neurotransmitter serotonin, having signaling roles in various biological processes, exhibiting antimicrobial, anti-inflammatory, and antioxidant properties, and serving as an important scaffold in pharmaceutical compounds [67, 68]. Additionally, it is produced by microorganisms during the degradation of organic matter and can be used as an indicator of microbial activity in environmental samples. The diverse roles of 1H-Indole in biological systems make it an important compound in biochemistry, pharmacology, and microbiology. 2,3-Dimethylquinolin-4(1H)-one is a chemical compound with potential biological significance. Its specific properties, interactions, and potential applications are important factors in determining its impact. Quinolinones, the class to which this compound belongs, have various biological effects, including antimicrobial, anticancer, anti-inflammatory, neuroprotective, enzyme inhibition, antioxidant, and metal chelation activities [69]. The biological significance of 2,3-Dimethylquinolin-4(1H)-one depends on its structure, functional groups, and specific research findings. These reported activities of 1H-Indole and 2,3-Dimethylquinolin-4(1H)-one may have contributed significantly to the neuroprotective impact of Jimson weed.

To further emphasize this report, the docking research findings collaborated with the *in vivo* study. Here, there was a strong indication that Ergost-5-en-3-ol and (24E)-24-N-Propylidenecholesterol effectively target caspase-3, IL-6, and TNF-α, which are pivotal in inflammation and cancer pathways. These compounds demonstrate binding energies similar to those of conventional neuroprotective drugs, (Amifostine) and (Leucovorin (Folinic Acid)) with some discrepancies. They may provide alternative treatment alternatives owing to their significant affinities, especially in targeting TNF-α and IL-6, which are pivotal in inflammation and cancer pathways. Ergost-5-en-3-ol engaged with critical residues in caspase-3, indicating a strong interaction with the protein's active site. It demonstrated comparable or even superior interactions relative to established pharmaceuticals, shown by the interaction of (24E)-24-N-Propylidenecholesterol with TNF-α *via* residues such as LYS and TYR, positioning it as a prospective option for prevention of neurotoxicity/inflammation associated with chemotherapeutic agents. The 2D and 3D interaction diagrams provide a comprehensive depiction of the interactions between the compounds and protein residues, validating that the ligands exhibited advantageous binding patterns. The compounds exhibited robust fitting into the binding pockets in the 3D view, notably Ergost-5-en-3-ol with caspase-3 and (24E)-24-N-Propylidenecholesterol with TNF-α, indicating a substantial degree of complementarity. The results indicate that the binding affinities and protein-ligand interactions of Ergost-5-en-3-ol and (24E)-24-N-Propylidenechol are notably favorable, particularly in comparison to Amifostine and Leucovorin. These natural substances have promise as alternatives or adjuncts to conventional pharmaceuticals, notably in addressing neurotoxicity/neuro-inflammatory mediators such as TNF-α and IL-6, which are pivotal in conditions including cancer, autoimmune illnesses, and chronic inflammation associated with chemotherapeutic agents.

Recent research has used molecular docking and bioinformatics methods to discover possible inhibitors for critical proteins implicated in inflammatory/tissue toxicity diseases. Caspase-3, an essential enzyme in apoptosis, has been explored as a target for anticancer drug development, with 1,2,4-Oxadiazole derivatives demonstrating favorable binding interactions [70]. Inhibitors of tumor necrosis factor-α (TNF-α) have been evaluated by cheminformatics and molecular dynamics simulations, identifying many compounds with significant binding affinities and advantageous ADMET profiles [71, 72]. Staurosporine and withanolide L were recognized as possible inhibitors of TNF-α and IL-6, respectively, in relation to rheumatoid arthritis [73]. A comparative bioinformatic study of TNF-α and TNF-β has shown conserved amino acid residues and binding grooves, with TNF-β exhibiting bigger surface cavities that might be beneficial for medication development [74]. These results provide significant insights for the development of more effective and selective inhibitors aimed at inflammatory proteins. Worthy of note is the structural similarity between these top 2 compounds and these referenced studied drugs.

The biological significance of Jimson weed (*Datura stramonium*) in the modulation of caspase-3, interleukin-6, and tumor necrosis factor-alpha (TNF-α) in neurotoxic rats is an area of interest in pharmacological and toxicological research. Jimson weed contains various alkaloids, including atropine, scopolamine, and hyoscyamine, which have been studied for their effects on the central nervous system. 1H-Indole is a heterocyclic organic compound. Indole is a key structural element in many natural and synthetic compounds, including tryptophan, an essential amino acid, and numerous pharmaceuticals and natural products. Its unique structure and reactivity make it an important building block in organic chemistry and drug discovery.

Caspase-3 is a key enzyme involved in apoptosis or programmed cell death, and its modulation by Jimson weed may have implications for regulating neuronal cell death and survival. Interleukin-6 and tumor necrosis factor-alpha are pro-inflammatory cytokines that play important roles in the immune response and neuroinflammation. Modulation of these cytokines by Jimson weed could potentially impact neuroinflammatory processes and neuronal damage. In the context of neurotoxicity, understanding how Jimson weed affects these molecular pathways could provide insights into its potential neuroprotective or neurotoxic effects. It's essential to note that Jimson weed can be highly toxic, and its use should be approached with caution. Research into its effects on molecular pathways such as caspase-3, interleukin-6, and tnf-α is crucial for understanding its potential therapeutic benefits and risks.

## CONCLUSION

MXT causes neurological impairments through oxidative stress, inflammation, and apoptosis. Jimson weed exerted neuroprotective properties *via* modulation of caspase-3, interleukin-6, and tumor necrosis factor-alpha(tnf-α) through binding of 1H-Indole and 2,3-Dimethylquinolin-4(1H)-one from ELEJW, reversing these neurological impairments in rats. Therefore, Jimson weed should be explored as a supportive therapy for the management of MXT-induced neurological derangements.

## AUTHORS’ CONTRIBUTIONS

The authors confirm their contribution to this paper as follow: conceptualization: EUA and ACF; methodology: EUA and ACF; resources: RK, MKG, and SS; supervision: SS and PV; validation: EUA and DEU; statistical analysis: EUA and DEU; writing - original draft: EUA, RK and MKG; writing - review & editing: EUA, MKG, RK, DEU, ACF, PV and SS. All Authors read and approved the manuscript for publication.

## Figures and Tables

**Fig. (5A):**
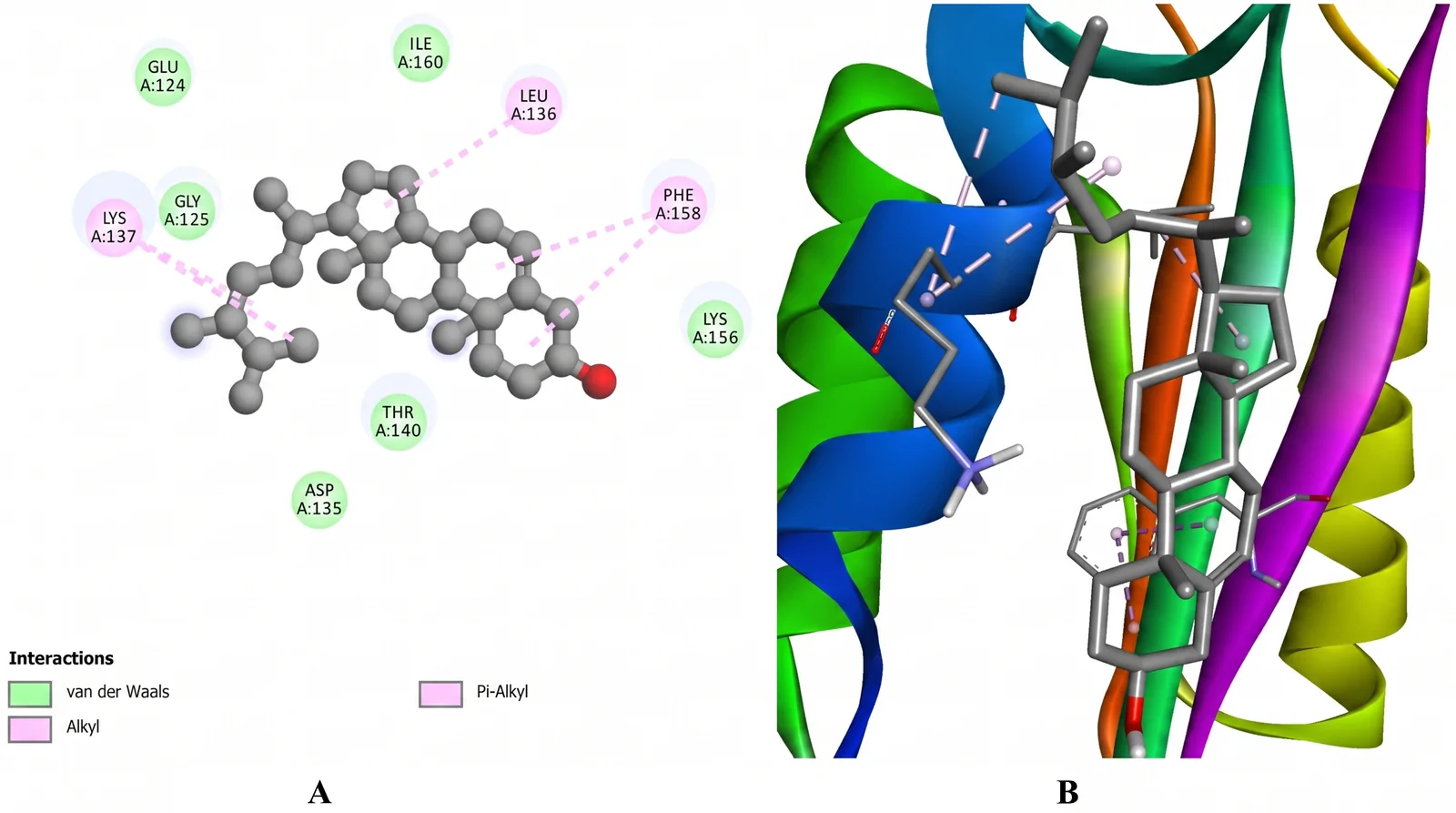
Ergost-5-en-3-ol and caspase-3 complex. **A**- 2D interaction showing amino acids residue and binding ligand **B**- 3D interaction showing the fitting of ligand into the binding pocket of caspase-3.

**Fig. (5B):**
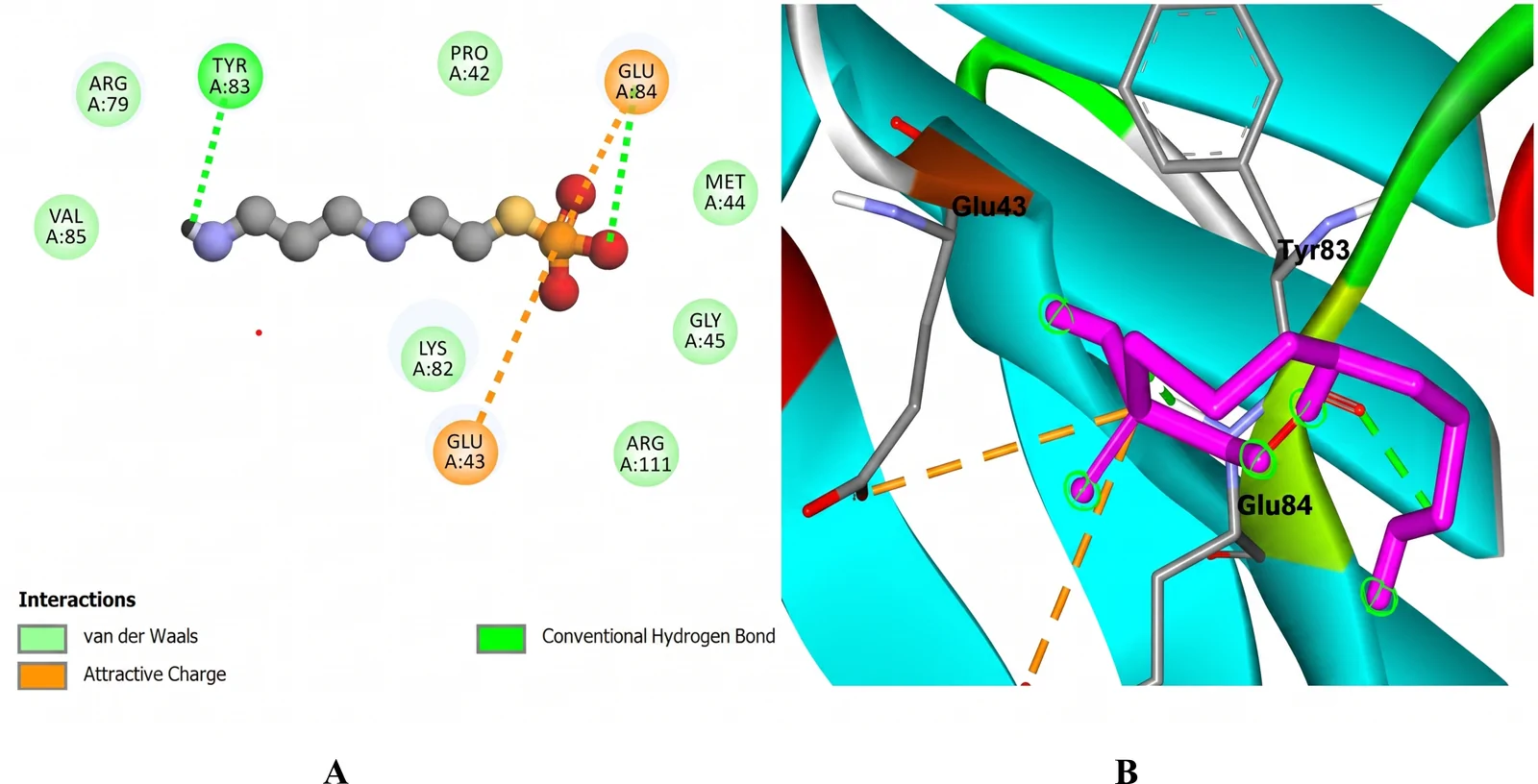
Standard [Amifostine (2141)] and caspase-3 complex. **A**- 2D interaction showing amino acids residue and binding ligand **B**- 3D interaction showing the fitting of ligand into the binding pocket of caspase-3.

**Fig. (6A):**
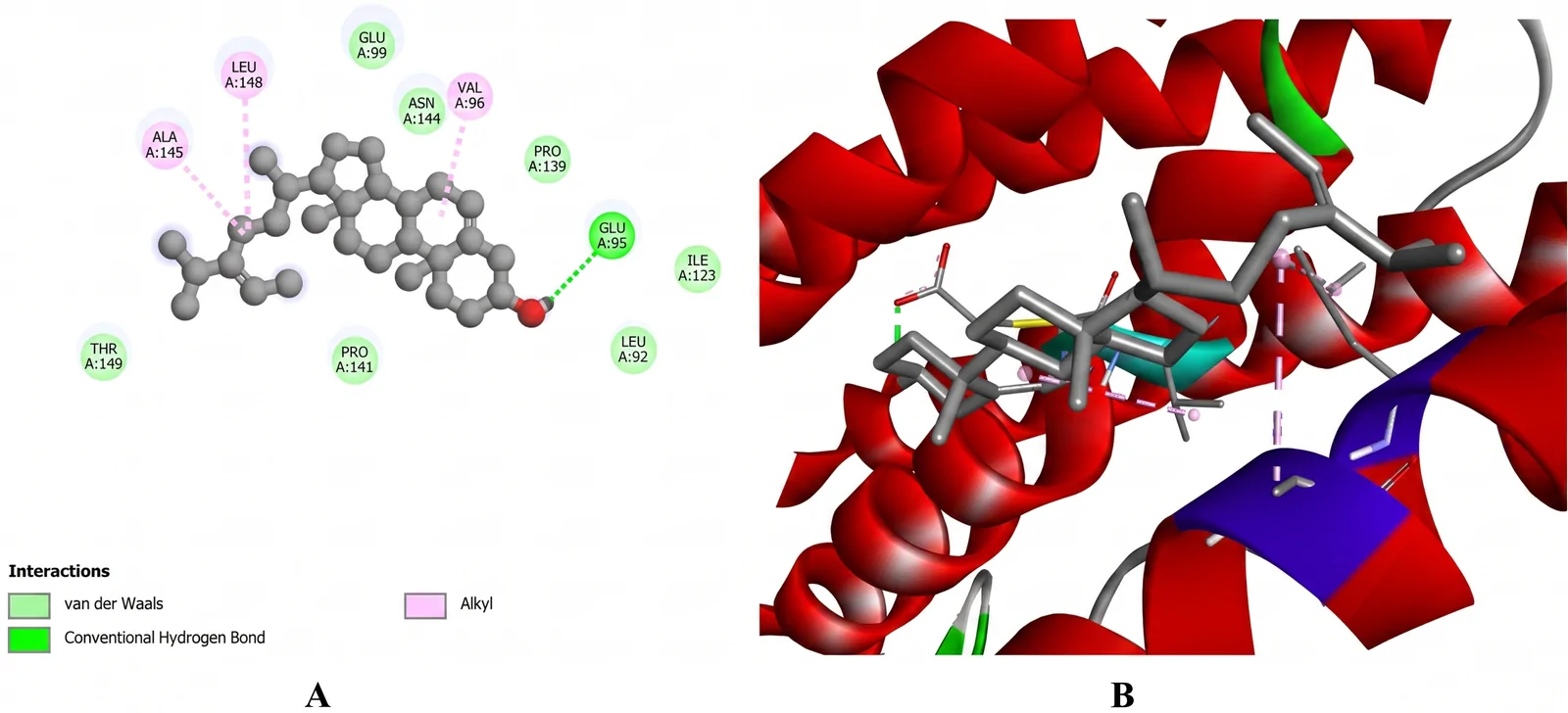
(24E)-24-N-Propylidenecholesterol and interleukin-6 (IL-6). **A**- 2D interaction showing amino acids residue and binding ligand **B**- 3D interaction showing the fitting of ligand into the binding pocket of IL-6.

**Fig. (6B):**
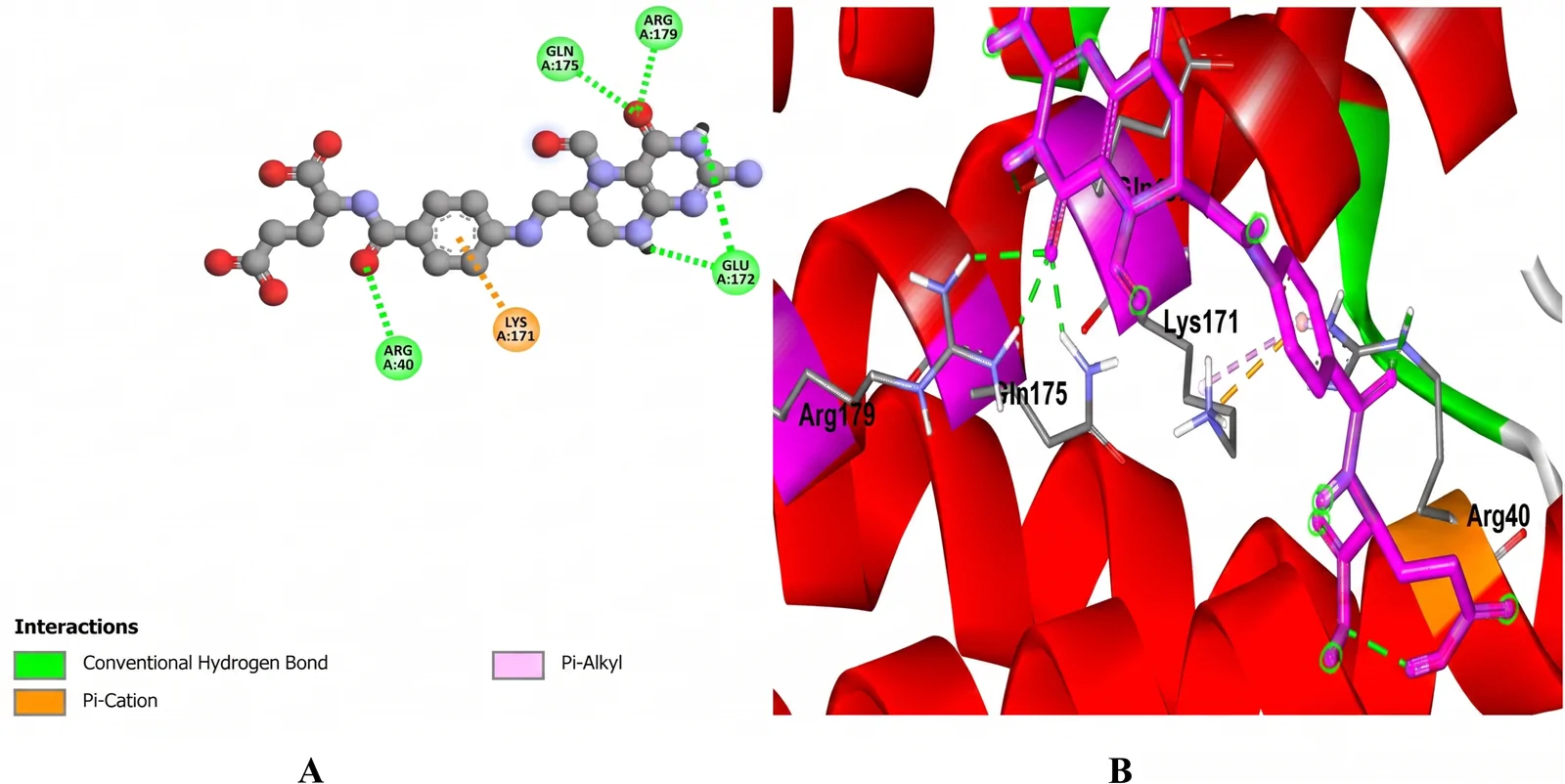
Standard [Leucovorin (Folinic Acid) ]and interleukin-6 (IL-6). **A**- 2D interaction showing amino acids residue and binding ligand **B**- 3D interaction showing the fitting of ligand into the binding pocket of IL-6.

**Fig. (7A):**
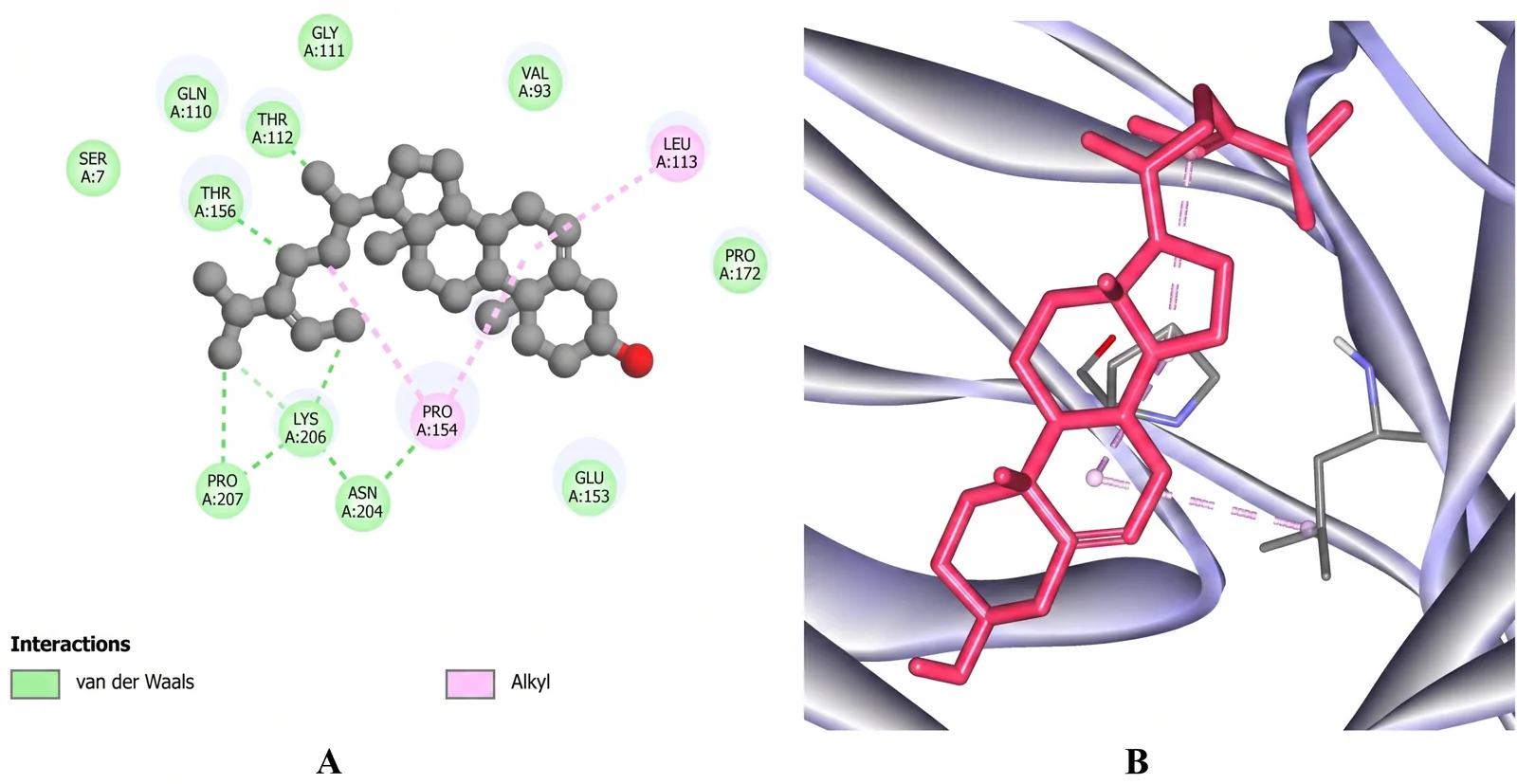
(24E)-24-N-Propylidenecholesterol and tumor necrosis factor-α (TNF-α). **A**- 2D interaction showing amino acids residue and binding ligand **B**- 3D interaction showing the fitting of ligand into the binding pocket of TNF-α.

**Fig. (7B):**
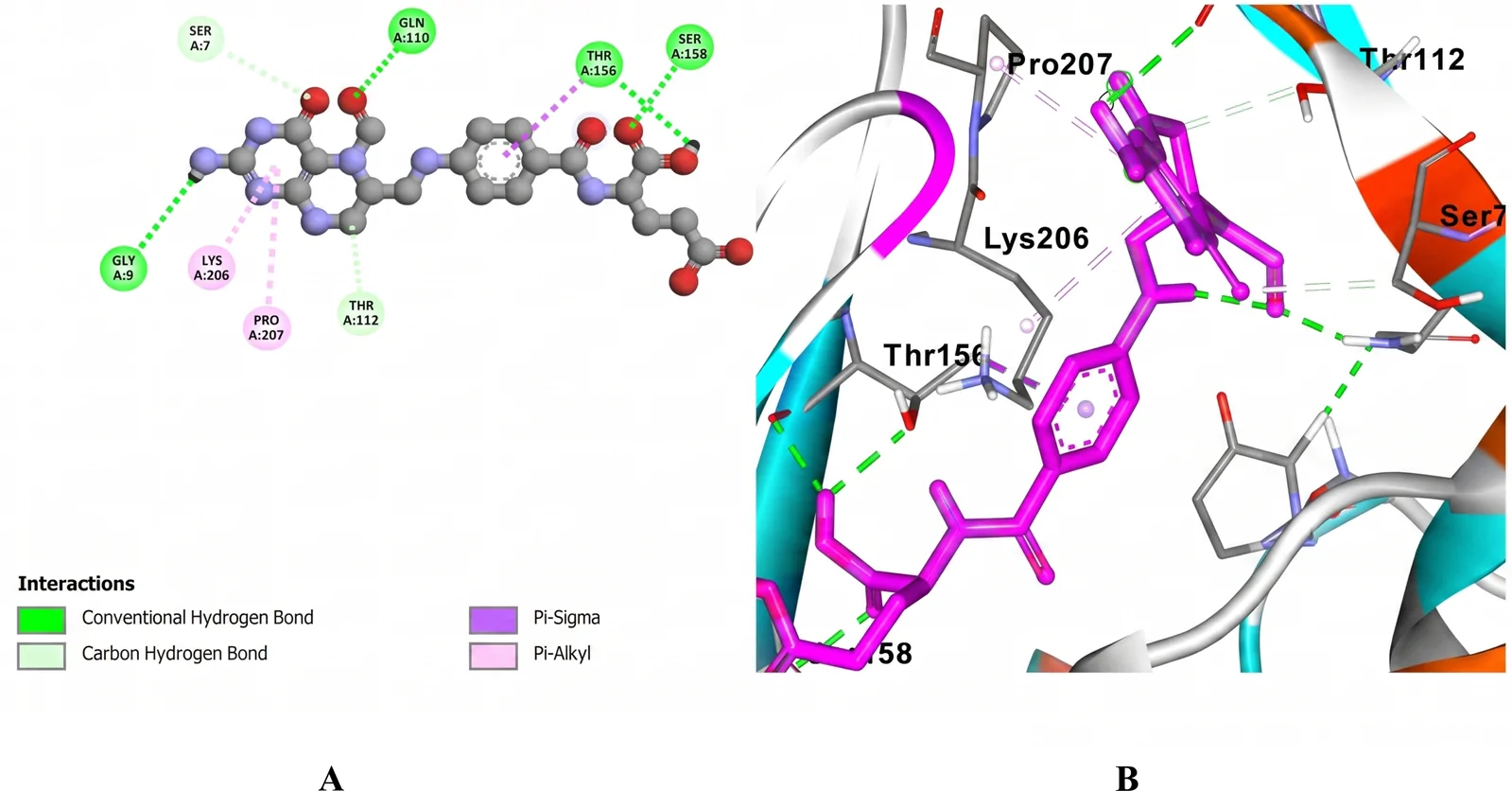
Standard [Leucovorin (Folinic Acid) and tumor necrosis factor-α (TNF-α). **A**- 2D interaction showing amino acids residue and binding ligand **B**- 3D interaction showing the fitting of ligand into the binding pocket of TNF-α.

**Table 1:** Docking of *Datura stramonium* compounds against selected protein targets (top nine compounds).

**S/N**	**Ligand**	**Protein Target**		
-	-	Caspase-3 PDB ID: 6X8I and	IL-6/IL-6Rα PDB ID: 5FUC	TNF-α PDB ID: 2AZ5
**1**	1H-Indole	-5.9	-6.6	-6
**2**	2,3-Dimethylquinolin-4(1H)-one	-5.9	-4.1	-4
**3**	Tetradecanoic Acid	-5.8	-4.5	-6.2
**4**	9,12,15-Octadecatrienoic acid	-5.7	-4.7	-4
**5**	Hexadecanoic Acid	-5.6	-4	-4.3
**6**	2-hydroxy-1-(hydroxymethyl)ethyl ester	-5.5	-3.5	- 4.7
**7**	(24E)-24-N-Propylidenecholesterol	-5.9	-6.9*	-7.1*
**8**	Gamma.-Tocopherol	-5.8	-4.1	-4.5
**9**	Ergost-5-en-3-ol	-6*	-4.5	-6.6
**10**	Standard drug (Amifostine)	-6.2	-	-
**11**	Standard drug (Leucovorin (Folinic Acid))	-	-6.9	-7.5

**Note:** *Compounds with the highest binding affinity towards protein targets; PDB ID: Protein Data Bank Identity.

**Table 2:** Pharmacokinetics and drug likeness properties of 1H-Indole and 2,3-Dimethylquinolin-4(1H).

-	-	-	**MEASURED ADMET PARAMETER**																			
**Compound**	PubChem ID	Mol. wt.	iLogP	HBA		HBD		Lipinski Violation		Ghose Violation	Verber Violation		HGIA		BBB		P-G S		CYP1A2 Inhibitor		CYP2C19 Inhibitor	
**1H-Indole**	798	117.15	1.43	0		1		0		3	0		High		+		-		-		+	
**2,3-Dimethylquinolin-4(1H)-one**	237396	173.21	1.98	1		1		0		0	0		High		+		+		-		+	
-	-	-	**MEASURED ADMET PARAMETER**																			
-	-	-	CYP2C9 Inhibitor		CYP2D6 Inhibitor		CYP3A4 Inhibitor		Caco-2	Carcinogenicity (binary)		Carcinogenicity (trinary)		Lead Likeness Violation		Synthetic Accessibility		TPSA		nrotb		Bioavailability
**1H-Indole**	798	117.15	-		-		-		+	-		Non-required		1		1		15.79		0		0.55
**2,3-Dimethylquinolin-4(1H)-one**	237396	173.21	-		-		-		+	-		Non-required		1		1.6		32.86		0		0.55

**Abbreviations:** HBD - H-bond Donors; HBA - H-bond Acceptors. Mol. Wt - molecular weight, HGIA - Human Gastro Intestinal Absorption BBB - Blood Brain Barrier P-GS P-Glycoprotein Substrate TPSA=Topological polar surface area, Number of Rotatable Bonds - nrotb.

## Data Availability

All data generated or analyzed during this study are included in this published article.

## LIST OF ABBREVIATIONS

MTX: Methotrexate
ELEJW: Ethanol Leaf extract of Jimson Weed
IL-6: Interleukin-6
IL-1: Interleukin-1
tnf-α: Tumour necrosis factor-alpha
ALL: Lymphoblastic Leukemia
SAM: S-adenosyl Methionine
MDA: Malondialdehyde
GSH: Reduced Glutathione
NO: Nitric Oxide
SOD: Superoxide Dismutase
GPx: Glutathione Peroxidase
CAT: Catalase
AchE: Acetylcholinesterase
ROS: Reactive Oxygen Species
